# PD‐L1 in oral squamous cell carcinoma: A key biomarker from the laboratory to the bedside

**DOI:** 10.1002/cre2.590

**Published:** 2022-05-20

**Authors:** Riccardo Nocini, Matteo Vianini, Ilaria Girolami, Luca Calabrese, Aldo Scarpa, Maurizio Martini, Patrizia Morbini, Stefano Marletta, Matteo Brunelli, Gabriele Molteni, Anil Parwani, Liron Pantanowitz, Albino Eccher

**Affiliations:** ^1^ Otolaryngology—Head and Neck Surgery Department University and Hospital Trust of Verona Verona Italy; ^2^ Department of Otolaryngology Villafranca Hospital Verona Italy; ^3^ Division of Pathology Central Hospital Bolzano Bolzano Italy; ^4^ Division of Otorhinolaryngology, Central Hospital Bolzano Bolzano Italy; ^5^ Department of Diagnostics and Public Health, Section of Pathology University of Verona Verona Italy; ^6^ Catholic University‐Fondazione Policlinico Universitario “A. Gemelli” IRCCS Rome Italy; ^7^ Department of Molecular Medicine Unit of Pathology, University of Pavia and Foundation IRCCS Policlinico S. Matteo Pavia Italy; ^8^ Department of Pathology, Wexner Medical Center Ohio State University Columbus Ohio USA; ^9^ Department of Pathology & Clinical Labs University of Michigan Ann Arbor Michigan USA; ^10^ Department of Pathology and Diagnostics University and Hospital Trust of Verona Verona Italy

**Keywords:** CD274, meta‐analysis, oral cancer, oral squamous cell carcinoma, PD‐L1, programmed death‐ligand 1

## Abstract

**Objectives and background**: Oral squamous cell carcinoma (OSCC) is a highly malignant disease with an increasing incidence. The need to improve therapeutic strategies for patients affected by OSCC is an urgent challenge. Currently, the advent of immunotherapy represents an important step toward this aim. Programmed cell death‐ligand 1 (PD‐L1), a membrane protein that can be expressed on tumor and inflammatory cells is a key biomarker whose expression is determined by means of immunohistochemistry and is necessary for selecting patients for immunotherapy. **Methods**: In this study, we review the methods of PD‐L1 assessment and outcomes achieved with immunotherapy in the treatment of OSCC patients. **Results**: Based on a meta‐analysis we demonstrate a lack of prognostic significance of PD‐L1 in OSCC. **Conclusions**: We also highlight unresolved issues including difficulties in standardizing PD‐L1 evaluation and discuss future opportunities such as leveraging digital pathology.

## 1 INTRODUCTION

Oral squamous cell carcinoma (OSCC) is the most common tumor of the oral cavity, with an increasing incidence worldwide, with up to 370,000 new cases per year (Sung et al., 2021). Although recent therapeutic opportunities have improved, OSCC is still responsible for up to 177,000 deaths annually, with a 5‐year overall survival (OS) rate of around 60% (Adamski et al., 2021).

Histologically, OSCC is composed of polygonal atypical squamous cells, which may have abundant eosinophilic cytoplasm and contain irregular nuclei, often with prominent nucleoli. Based on cellular atypia and structural architecture, OSCC is graded into well, moderately, and poorly differentiated tumors. Tumor grading, as assessed by pathologists using light microscopy, has direct implications for prognostic stratification, with poorly differentiated tumors usually associated with an increased risk of disease recurrence and death.

Programmed cell death‐ligand 1 (PD‐L1) is a transmembrane protein that can be expressed on neoplastic cells and tumor‐infiltrating immune cells. Its interaction with programmed cell death protein 1 (PD‐1), which is usually expressed on activated T lymphocytes, causes the inactivation of the self/cell‐mediated immune response against tumor cells (Lenouvel et al., 2020). Recent evidence has pointed out that the PD‐1/PD‐L1 axis is typically altered in a significant proportion of OSCC cases, rendering this tumor type a promising candidate for immune checkpoint inhibitors, with the selective blockade of the PD‐L1/PD‐1 axis. Such targeted immunotherapy can be successfully achieved through the use of monoclonal antibodies directed either against PD‐L1 (e.g., Atezolizumab) or against PD‐1 (e.g., Pembrolizumab). By restoring antitumor adaptive immunity, immune checkpoint inhibitors have become one of the most important actors in the rapidly changing landscape of cancer immunotherapy (Paver et al., 2021).

**Figure 1:**
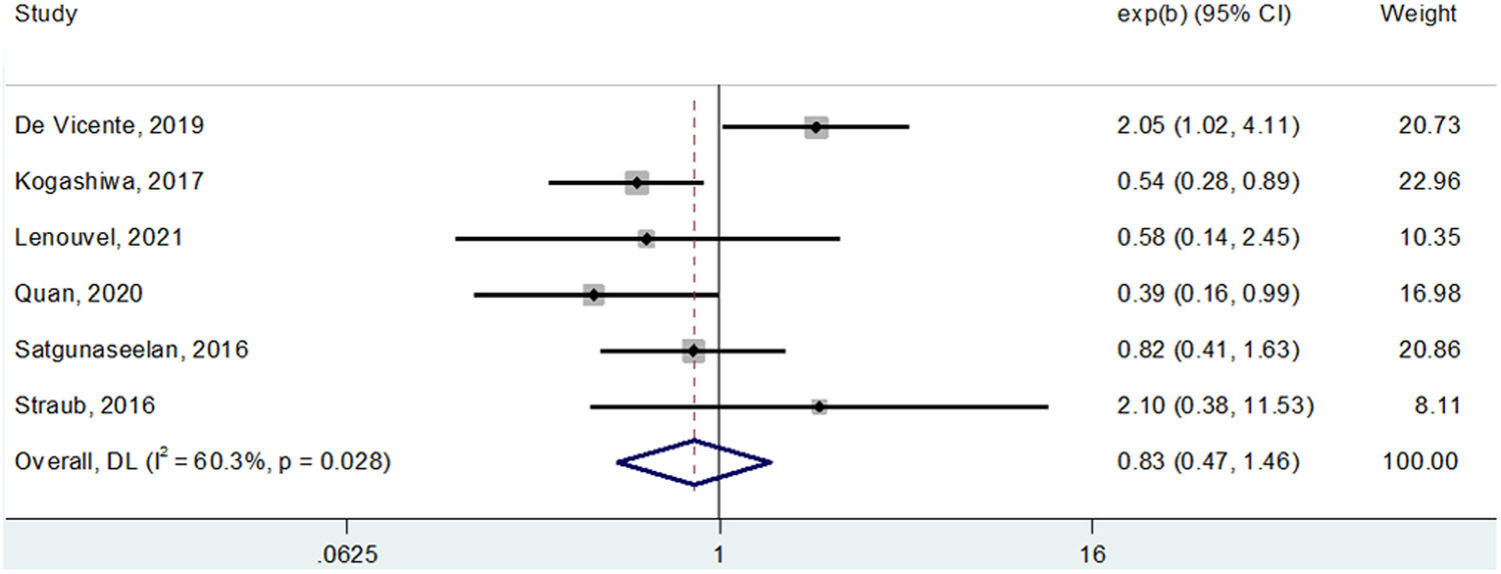
Forest plot showing the impact of PD‐L1 expression on overall survival in patients with oral squamous cell carcinoma. As shown by the direct contact of the summarizing box with the “1‐line,” statistical significance is not reached. PD‐L1, programmed cell death‐ligand 1.

The expression of PD‐L1 can be evaluated on both tumor cells and immune cells with immunohistochemistry (IHC), which is assessed by pathologists examining formalin‐fixed paraffin‐embedded tissues using light microscopy (Figure 1). Positive staining and the patterns of PD‐L1 immunoexpression represent one of the most important indicators for correctly selecting patients' eligibility for immunotherapy (Luchini et al., 2019). PD‐L1 is preferred to PD‐1 as a predictive biomarker of immunotherapy response since its expression is more reliably detected with IHC and unlike PD‐1, which is assessed only in inflammatory cells, PD‐L1 can be detected on both tumor and inflammatory cells.

In the present work, we review the fundamental role of PD‐L1 in OSCC, providing insight into recent advances and future perspectives of this crucial biomarker from the laboratory to the bedside. A meta‐analysis regarding the prognostic role of PD‐L1 in OSCC is also performed.

### 1.1 PD‐L1 assessment with IHC

The manual assessment of PD‐L1 via IHC remains a challenge for pathologists. There are several commercially available clones of the anti‐PD‐L1 antibody, each associated with different modalities of evaluation and thresholds of positivity. The evaluation of PD‐L1 in neoplastic cells must be performed in tumor areas comprising at least 100 viable cells and only a membranous staining pattern should be considered to represent positivity (Chen et al., 2019; Luchini et al., 2019). As for the evaluation of PD‐L1 in inflammatory cells, pathologists should only assess those inflammatory cells infiltrating tumor areas or in close relationship with tumor cells (so‐called “tumor‐infiltrating” immune cells). The inflammatory cell population is usually composed of lymphocytes (mainly cytotoxic CD8‐positive T lymphocytes) and macrophages. Unlike tumor cells, tumor‐infiltrating inflammatory cells are considered to be positive if any PD‐L1 staining is evident, independent of their staining pattern (Ventana PD‐L1 [SP142] Assay, 2019).

Another unsettled issue pertains to the different scoring systems used for quantifying PD‐L1 staining patterns in different tumors. Along this line, two different scores have been recently introduced, and currently, they represent the gold standard for PD‐L1 evaluation in certain solid malignancies, including gastrointestinal cancer, non‐small cell lung cancer, as well as head and neck squamous cell carcinoma (Cohen et al., 2019; Paolino et al., 2021). These two scores are: (1) tumor proportion score (TPS), which represents the proportion (%) of PD‐L1 positive tumor cells relative to the total number of viable tumor cells × 100; and (2) combined positive score (CPS), which represents the number of all PD‐L1‐positive cells (neoplastic and inflammatory cells) relative to the number of all viable tumor cells × 100 (Paolino et al., 2021). Additional scores have been introduced for other solid tumors, including triple‐negative breast cancer and urothelial carcinoma. These extra scoring systems are: (1) immune cell score (IC), which only takes into account immune cells and is expressed as a percentage of the tissue area occupied by PD‐L1 positive immune cells to the total tissue area; and (2) tumor cell score (TC), which is a percentage of the PD‐L1‐positive tumor cells to the total number of tumor cells, similar to the TPS (Cohen et al., 2019; Crosta et al., 2021; Paolino et al., 2021). These different scores have been developed for the evaluation of PD‐L1 staining patterns with specific clones. For example, TPS and CPS are utilized in association with the clone 22C3 and IC/TC for the clone SP142. Currently, for immunotherapy eligibility of patients with OSCC, the score that is recommended in clinical practice is CPS for evaluating the 22C3 clone.

Given the cost of different platforms needed for preparation, not all PD‐L1 clones can be made simultaneously available in most hospitals, which may further impede IHC evaluation. Furthermore, subjectivity may exist when manually assessing PD‐L1 staining. Indeed, some recent studies have reported medium‐to‐low interobserver agreement in this regard among different pathologists (Crosta et al., 2021; Das et al., 2021; Dong et al., 2021; Pang et al., 2021). Of note, encouraging results showing moderate‐to‐high concordance have been recently reported for head and neck squamous cell carcinoma, based largely on the use of a standardized CPS score (Cerbelli et al., 2021; Girolami et al., 2021). Finally, the evaluation of PD‐L1 expression in small biopsy material can be very difficult and may generate false results, especially due to the heterogeneity of PD‐L1 expression in tissue samples (Kwon et al., 2018; Paolino et al., 2021). To overcome the aforementioned issues and have the field move toward more standardized evaluation, new approaches that leverage digital pathology (e.g., computer‐assisted image analysis) may better support pathologists when performing this difficult task.

### 1.2 PD‐L1 expression in precursor lesions and during OSCC tumor progression

PD‐L1 expression increases when oral precancerous lesions progress to invasive OSCC. The analysis of a cohort of 49 oral lesions by Dave et al. (including 20 nonprogressing dysplasia cases, 19 progressing dysplasia, and 10 invasive OSCC) showed a significant increase in PD‐L1 expression during OSCC progression (Girolami, Pantanowitz, Munari, et al., 2020). Similar findings were reported by other authors involving cases of oral leukoplakia with a high risk of malignant transformation (Girolami et al., 2020; Gurizzan et al., 2021; Kim et al., 2019). The increased expression of PD‐L1 during OSCC carcinogenesis is accompanied by profound modifications in the tumor microenvironment. Along this line, Das et al. (2021) described a crucial role of interferon receptor signaling in tumor growth by activating immune evasion mechanisms. Thus, PD‐L1 expression provides direct evidence of OSCC immune evasion toward malignant transformation and progression. All these findings suggest that PD‐L1 can be used as a helpful tool to recognize high‐risk progressing lesions, and have led to the development of in vitro and animal studies (Dong et al., 2021), followed by human clinical trials (Lee et al., 2021), to investigate the potential role of immunotherapy for preventing the malignant transformation of oral precancerous lesions. The overall results of these exploratory studies demonstrate the feasibility of immunotherapy in preventing the evolution of squamous dysplasia. Thus, in selected cases, restoring the activity of the immune system through the administration of checkpoint inhibitors may play an important role in preventing OSCC occurrence (Lee et al., 2021).

### 1.3 PD‐L1 is an unreliable marker for OSCC prognostic stratification

Given the increased expression of PD‐L1 during the malignant transformation of OSCC, several studies have tried to establish whether this biomarker was also an indicator of poor prognosis in this tumor type. An adverse prognostic value of PD‐L1 has already been demonstrated in other cancers, such as non‐small cell lung cancer, pancreatic undifferentiated carcinoma, thyroid, and prostate cancer (Lenouvel et al., 2020, 2021; Luchini et al., 2018; Shen et al., 2021). For OSCC, there are conflicting results, with some reports denoting PD‐L1 expression as an indicator of poor outcome and other studies supporting an opposing conclusion. While published meta‐analyses reveal the limited prognostic impact of PD‐L1, these pooled studies reported conflicting results (Lenouvel et al., 2020; Luchini et al., 2019).

To shed light on this complex scenario, we performed our own systematic review and meta‐analysis taking into account all published studies on this topic.

## 2 METHODS

The review question was built upon a Population, Index, Comparator, Outcome (PICO) frame. The “population” is represented by patients with OSCC, “index” by the expression of PD‐L1 considered positive as defined in a single study in “comparison” to negative expression, and “outcome” incorporates survival measures of OS and disease‐free survival (DFS). The inclusion criteria derived from this framework were: (i) both prospective and retrospective studies investigating PD‐L1 expression in samples procured from primary and naive OSCC patients; and (ii) studies that reported survival indexes based on calculating multivariable hazard ratio (HR) and its 95% confidence interval (95% CI) for at least OS and/or DFS. Our meta‐analysis adhered to existing guidelines, including Meta‐analyses Of Observational Studies in Epidemiology (MOOSE) guidelines (Stroup, 2000), Preferred Reporting Items for Systematic Reviews and Meta‐Analyses (PRISMA), and the Newcastle‐Ottawa Scale (NOS) statement (Liberati et al., 2009; Mattox et al., 2017). Studies not dealing with OSCC, not reporting PD‐L1 positive and negative expression, or not reporting survival data in an extractable manner were excluded, as were studies represented by abstracts only or other article types, such as letters, reviews, animal and cell culture studies, and case reports. A literature search was performed using PubMed and Scopus without language restriction, from database inception until 23 October, 2021 with the following search strategy: (“PD‐L1” OR “PDL1” OR “CD274” OR “B7‐H1” OR “Programmed Death Ligand 1” OR “Programmed Death‐Ligand 1”) AND (“oral cancer” OR “oral squamous” OR “oral squamous cell” OR “oral carcinoma”). Two authors (R. N. and M. V.) separately screened the title and abstracts for potential inclusion; disagreement was resolved by consensus. The full text of potentially eligible articles was retrieved and evaluated by two authors to verify inclusion criteria and the quality of these studies. By applying the inclusion criteria, 12 studies were selected (Table 1) (Cho et al., 2011; de Vicente et al., 2019; Kogashiwa et al., 2017; Lenouvel et al., 2021; Mattox et al., 2017; Oliveira‐Costa et al., 2015; Pang et al., 2021; Quan et al., 2020; Satgunaseelan et al., 2016; Shen et al., 2021; Straub et al., 2016; Wu et al., 2021). Data extracted included: country and authors of each study, number of patients, clone of PD‐L1 used, cut‐off for positivity applied, and the HR for OS and DFS reported in the study. Pooled HR was calculated using DerSimonian‐Laird random‐effects models and heterogeneity across studies was assessed by the I2 metric.

**Table 1:** Summarizing table of studies reporting HR data regarding the impact on survival indexes of PD‐L1 expression in oral squamous cell carcinoma

Authors, years	Country	No. of patients	Detection method	Clone of PD‐L1 antibody	Cut‐off for positivity	HR overall survival (95% CI)	HR disease‐free survival (95% CI)
Cho (2011)	South Korea	45	IHC	ab82059	Score >2	1.10 (043−2.82)	NA
Oliveira‐Costa (2015)	Brazil	96	IHC	ab28753	>5% of tumor cells	0.43 (0.29−0.63)	NA
Satgunaseelan (2016)	Australia	217	IHC	E1L3N	>5% of tumor cells	NA	0.82 (0.41−1.63)
Straub (2016)	Germany	80	IHC	E1L3N	>5% of tumor cells	2.94 (0.20−43.94)	2.10 (0.38−11.53)
Mattox (2017)	USA	53	IHC	5H1	>1% of tumor and/or immune cells	1.62 (0.22−11.73)	NA
Kogashiwa (2017)	Japan	84	IHC	SP142	>5% of tumor cells	0.26 (0.10−0.65)	0.54 (0.28−0.89)
De Vicente (2019)	Spain	125	IHC	22C3	>10%	NA	2.05 (1.02–4.11)
Quan (2020)	China	88	IHC	EPR19759	>25% tumor cells; >10% immune cells	NA	0.39 (0.16−0.99)
Adamski (2021)	Poland	109	IHC	E1L3N	>10% tumor cells; >20% immune cells	2.51 (1.98−5.29)^a^	NA
Lenouvel (2021)	Spain	55	IHC	22C3	TPS >5%	1.15 (0.48−2.73)	0.58 (0.14−2.45)
Wu (2021)	China	150	TIMER	NA	NA	1.67 (1.10−2.50)	NA
Subramaniam (2021)	India	64	IHC	NS	TILs >1%	0.59 (0.10−1.04)^b^	NA

Abbreviations: CI, confidence interval; HR, hazard ratio; IHC, immunohistochemistry; PD‐L1, programmed cell death‐ligand 1; TIMER, tumor immune estimation resource.

^a^ Specific data in squamous cell carcinoma of the tongue and the floor of the mouth.

^b^ Data on TILs only.

## 3 RESULTS AND DISCUSSION

**Figure 2:**
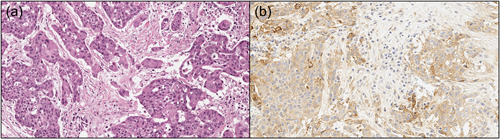
A classic example of a case of invasive oral squamous cell carcinoma, poorly differentiated, showing diffuse positivity for PD‐L1. (a) Hematoxylin & eosin stain, original magnification ×20; (b) PD‐L1 immunostaining with 22C3 antibody, 90% TPS, 90 CPS. CPS, combined positive score; PD‐L1, programmed cell death‐ligand 1; TPS, tumor proportion score.

**Figure 3:**
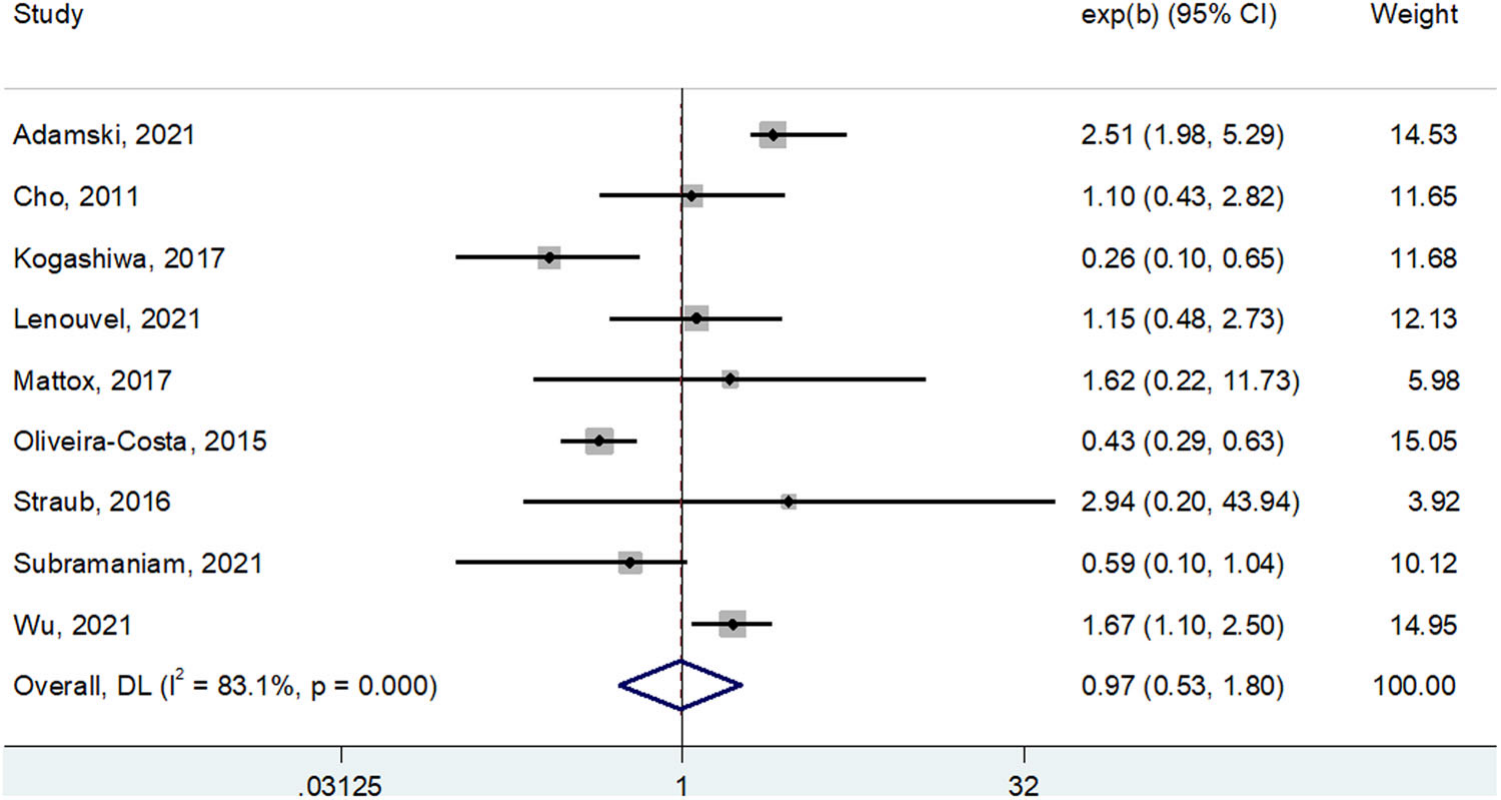
Forest plot showing the impact of PD‐L1 expression on disease‐free survival in patients with oral squamous cell carcinoma. As shown by the direct contact of the summarizing box with the “1‐line,” statistical significance is not reached. PD‐L1, programmed cell death‐ligand 1.

The main result of this meta‐analysis, as also shown in Figure 2 (OS) and Figure 3 (DFS), is a definitive demonstration, using the most powerful survival indexes (HR from multivariable analysis), that PD‐L1 cannot be used as a significant prognosticator for OSCC patients (HR for OS: 0.97, 95% CI: 0.53−1.80; HR for DFS: 0.83, 95% CI: 0.47−1.46). Of interest, the study published by Adamski et al. (2021) indicated a potential significant association between PD‐L1 expression on tumor cells and a poor prognosis for squamous cell carcinoma of the tongue and the floor of the mouth, but not of other oral compartments (Shen et al., 2021). It remains to be determined if this conflicting literature is at all dependent on the aforementioned complexities about PD‐L1 assessment in OSCC (e.g., varying clones, scores, and thresholds for positive staining). For example, in the 12 manuscripts selected for our meta‐analysis, 7 different clones were used (Table 1). With the recent approval of Federal Drug Administration (FDA) and European Medicines Agencies (EMA) guidelines, there may be more standardization, which would in turn benefit the appropriate selection of immunotherapy.

### 3.1 PD‐L1 for OSSC in clinical practice

Immunotherapy‐based approaches for the treatment of OSCC are already playing an important role in clinical practice. The PD‐1 inhibitor nivolumab received FDA approval for patients progressing with the disease after first‐line platinum‐based therapy, based on findings of the “Checkmate‐141” trial (Ferris et al., 2018). Based on the published findings of two distinct clinical trials, named “KEYNOTE‐012” and “KEYNOTE‐040,” the PD‐1 inhibitor pembrolizumab was officially approved by the FDA and EMA for the treatment of recurrent and metastatic head and neck squamous cell carcinoma, in those cases with a TPS >50% and evaluated with the 22C3 clone (pharmDx/Agilent Technologies, Inc.) at IHC (Cohen et al., 2019; Mehra et al., 2018).

A subsequent randomized, open‐label, Phase 3 clinical trial named “KEYNOTE‐048” demonstrated that pembrolizumab was associated with improved OS compared to the alternate standard of care for patients with head and neck squamous cell carcinoma (Burtness et al., 2019), thereby indicating that pembrolizumab monotherapy is an appropriate first‐line treatment for PD‐L1‐positive (i.e., CPS > 1) recurrent and metastatic tumors (Borcoman et al., 2021). Subsequent analysis demonstrated that the CPS score has increased sensitivity compared to the TPS score (de Vicente et al., 2019). These trials have also shown that PD‐L1 expression is associated with an increased objective response rate in patients with CPS ≥ 1 and with a better response for CPS value ≥ 20 (Burtness et al., 2019). Indeed, the EMA approved pembrolizumab to be used both as monotherapy or in combination with chemotherapy, as first‐line treatment for recurrent and metastatic head and neck squamous cell carcinoma in patients whose tumors express PD‐L1 with a CPS ≥ 1, regardless of the test (antibody and IHC platform) used. Therefore, a predictive role of PD‐L1 expression has been established, since CPS categorization has clinical and therapeutic implications, and a proportion of negative patients (CPS ≤ 1) of around 15% is expected (Burtness et al., 2019; de Vicente et al., 2019). Moreover, at CPS ≥ 20 an enhanced therapeutic response is expected, thus reinforcing the predictive role of the CPS value.

Recently, the important oncology concept referred to as hyperprogressive disease (HD) was introduced, based on the observation of different response patterns to immunotherapy during cancer treatment (specifically, rapid tumor progression after the initiation of immunotherapy) (Kim et al., 2019). HD has been observed across various types of cancers, including OSCC, and has been associated with poor survival (Kim et al., 2019). Of note, IHC positivity for PD‐L1 is inversely correlated with HD, further highlighting the importance of striving for a standardized assessment of this biomarker.

## 4 CONCLUSION AND FUTURE PERSPECTIVES

The main highlights gathered in this study are summarized in Table 2. The most important theme to emerge is the need for standardized guidelines for PD‐L1 evaluation that clearly elucidate how best to handle intratumor heterogeneity of PD‐L1 expression, subjectivity in IHC evaluation, and the utilization of different clones with different scores. Future perspectives for advancing the management of OSCC patients based on PD‐L1 assessment could benefit from advances in digital pathology‐based tools, such as automated computer‐assisted quantitative image analysis, for supporting IHC evaluation and thereby improving the selection of patients for immunotherapy. Interestingly, a recent study has indicated that high PD‐L1 expression in OSCC may even be predicted with a similar analysis of radiology images (Tekiki et al., 2021). Finally, based on the finding of PD‐L1 expression in precancerous lesions and invasive OSCC, new therapeutic strategies based on immune checkpoint inhibitors may begin to be applied in selected cases with a high risk of malignant transformation.

**Table 2:** Summary of the main findings of the current study

Evidence	Main findings
Prognostic value of PD‐L1	Current evidence does not support the role of PD‐L1 expression as a significant prognosticator for OSCC patients (HR for OS: 0.97, 95% CI: 0.53−1.80; HR for DFS: 0.83, 95% CI: 0.47−1.46).
Predictive value of PD‐L1	Trials support that PD‐L1 expression is associated with an increased objective response rate in patients with CPS ≥1, with better response with CPS value ≥20.
Limitations of studies available in the literature	High heterogeneity of studies in terms of PD‐L1 clone and platform used.
	Different scoring systems for defining positivity.
	Suboptimal investigation of effects of previous therapy on PD‐L1 expression.
Future directions	Standardization of clones and scoring systems to have more homogeneous data.
	Best selection of patients.
	Aid coming from artificial intelligence tools on digital slides to evaluate PD‐L1 expression.

Abbreviations: CI, confidence intervals; CPS, combined positive score; DFS, disease‐free survival; HR, hazard ratio; OS, overall survival; OSCC, oral squamous cell carcinoma; PD‐L1, programmed cell death‐ligand 1.

## AUTHOR CONTRIBUTIONS

All authors have participated in the conception and design or analysis and interpretation of the data. All authors contributed to the drafting of the manuscript and approved the final version of the manuscript. The study was supported by a fund from Barone Rossi and Community of Albaredo d'Adige.

## CONFLICTS OF INTEREST

The authors declare no conflicts of interest.

## Data Availability

Data sharing is not applicable to this article as no new data were created or analyzed in this study.
